# 3D tissue-engineered lung models to study immune responses following viral infections of the small airways

**DOI:** 10.1186/s13287-022-03134-1

**Published:** 2022-09-07

**Authors:** Taylor Do, Lilly Synan, Gibran Ali, Heather Gappa-Fahlenkamp

**Affiliations:** Edward Bartlett Chair, School of Chemical Engineering, Oklahoma State University, 420 Engineering North, Stillwater, OK 74078 USA

**Keywords:** Tissue-engineered lung model, Respiratory syncytial virus, Epithelial cells, Endothelial cells, Myeloid cells, Scaffolds

## Abstract

Small airway infections caused by respiratory viruses are some of the most prevalent causes of illness and death. With the recent worldwide pandemic due to the severe acute respiratory syndrome coronavirus 2 (SARS-CoV-2), there is currently a push in developing models to better understand respiratory diseases. Recent advancements have made it possible to create three-dimensional (3D) tissue-engineered models of different organs. The 3D environment is crucial to study physiological, pathophysiological, and immunomodulatory responses against different respiratory conditions. A 3D human tissue-engineered lung model that exhibits a normal immunological response against infectious agents could elucidate viral and host determinants. To create 3D small airway lung models in vitro, resident epithelial cells at the air–liquid interface are co-cultured with fibroblasts, myeloid cells, and endothelial cells. The air–liquid interface is a key culture condition to develop and differentiate airway epithelial cells in vitro. Primary human epithelial and myeloid cells are considered the best 3D model for studying viral immune responses including migration, differentiation, and the release of cytokines. Future studies may focus on utilizing bioreactors to scale up the production of 3D human tissue-engineered lung models. This review outlines the use of various cell types, scaffolds, and culture conditions for creating 3D human tissue-engineered lung models. Further, several models used to study immune responses against respiratory viruses, such as the respiratory syncytial virus, are analyzed, showing how the microenvironment aids in understanding immune responses elicited after viral infections.

## Introduction

Lower respiratory infections are the most prevalent cause of illness in the modern world. New strains of respiratory viruses are continuously emerging, such as the recent SARS-CoV-2 pandemic. According to the Centers for Disease Control and Prevention (CDC), from 1999 to 2018 in the USA alone, chronic lower respiratory diseases accounted for around 2.75 million deaths, making it the fourth leading cause of death [1]. Some common viruses affecting human lungs include influenza, parainfluenza, respiratory syncytial virus (RSV), adenovirus, enterovirus, and coronavirus. SARS-CoV-2 has led to over 5.878 million deaths worldwide since its outbreak [2]. While many that are infected show mild-to-moderate symptoms, these viruses can cause more serious complications such as pneumonia, which can be fatal. Since these viruses infect and replicate through healthy cells, understanding the human immune response is the key in treating infections. By understanding the immune response of cells in the lower respiratory system, the long-term effects of viruses can be halted and eliminated.

Respiratory virus strains can elicit a unique physiological response in individuals. Previous work with various in vitro respiratory models has provided insight into respiratory viral infections and the resulting immune response. In general, respiratory viruses infect airway epithelial cells before migrating to blood macrophages and dendritic cells [3]. The pro-inflammatory cytokines upregulated during infection include IL-6 and TNF-α, and highly upregulated chemokines MIP-1α, MCP-1, and RANTES [4]. Further, respiratory viruses can trigger an immune response that leads to a “cytokine storm” where uncontrolled levels of pro-and anti-inflammatory cytokines are produced [5]. In turn, this can cause physiological damage to the alveolar barrier function and ultimately lead to flooding of the alveolar airspace with fluid, erythrocytes and leukocytes, and decreased gas exchange, ultimately causing severe respiratory insufficiency and death [6, 7].

Viral infections affect several cell types and result in complex cellular responses that are not completely understood. Recently, researchers have been shifting their interest toward creating 3D human tissue-engineered lung models (3D-HTLM) to study these complex cellular responses. A 3D in vitro HTLM can provide a physiological environment that mimics in vivo conditions while minimizing variability commonly seen in other models like tissue explants and animal models. These in vitro 3D-HTLM consist of multiple cell types grown on scaffolds to capture pathogenesis and tropism of virus strains and cellular responses. The 3D scaffolds provide a microenvironment for primary human cells to differentiate and behave more like in vivo conditions. This paper will review the most commonly used cell types, scaffold materials, and culture conditions used to create 3D in vitro HTLM to study respiratory viruses and immune responses.

## Small airway cells used in 3D tissue-engineered lung models

**Fig. 1:**
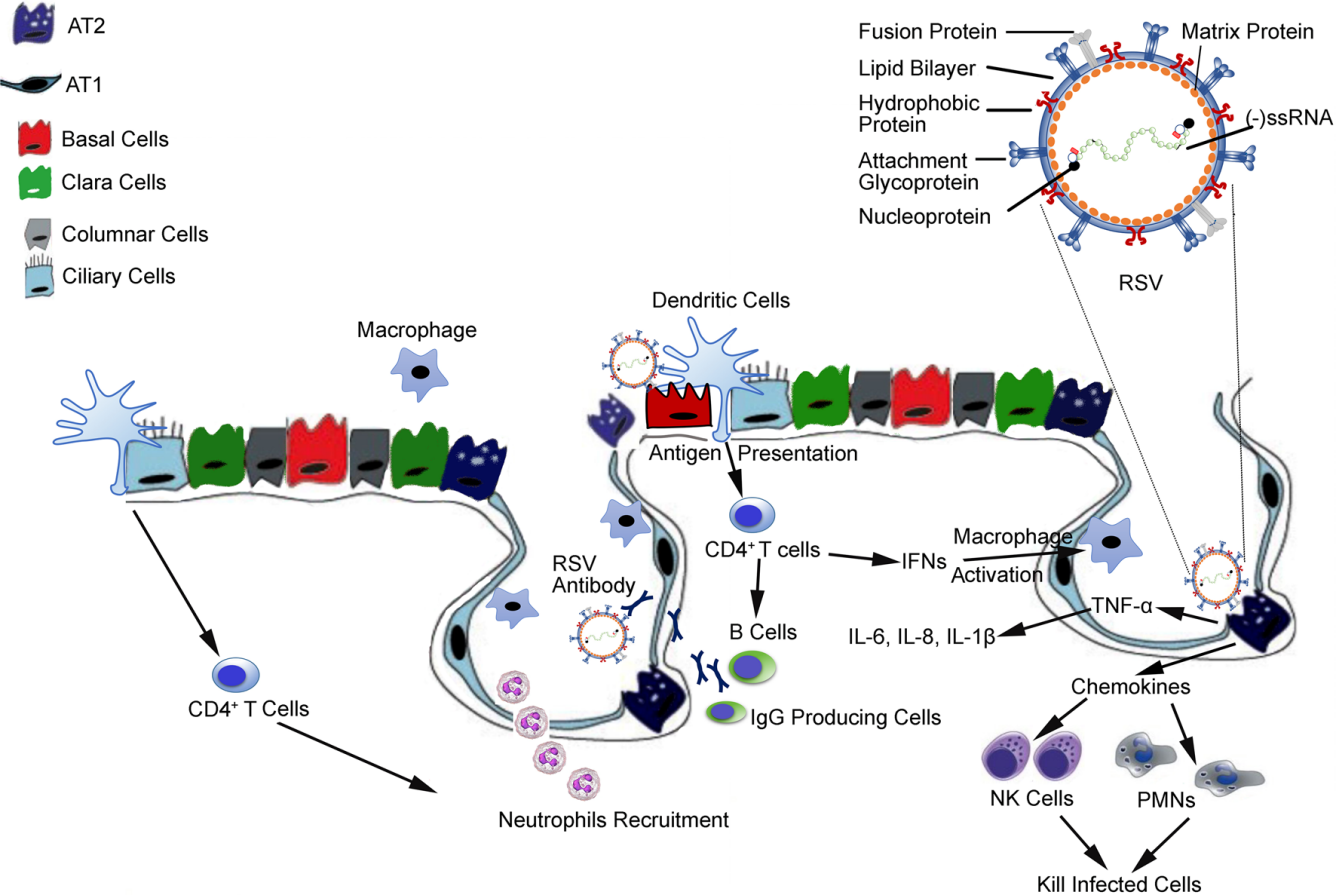
Immune responses to RSV infection. Infection of RSV particle causes the release of cytokines and chemokines, resulting in recruitment of immune cells. Chemokines released from alveolar type 2 (AT2) cells signal for natural killer (NK) cells and polymorphonuclear (PMN) leukocytes to kill infected cells as part of a nonspecific immune response. AT2 cells also release TNF-α, IL-6, IL-8, and IL-1β, causing activation of dendritic cells and macrophages and the recruitment of CD4^+^ T cells. These T cells release interferons (IFNs) that activate macrophages and help activate B cells which are required for the development of RSV antibodies. CD4^+^ T cells also recruit neutrophils from the blood to alveolar air spaces

The airway epithelium is considered a major part of the respiratory system in orchestrating the inflammatory and immune responses. Epithelial cells act together with resident cells and recruit immune cells to regulate alveolar immunity. Macrophages derived from fetal monocytes are the most abundant type of immune cells located in the airway lumen and self-maintained locally throughout life [8]. This immune response is characterized by the differentiation of monocytes into alveolar macrophages, which serve as a first-line of defense against invading viruses [9]. Natural killer cells (NK) activated by interferons not only destroy virally infected cells but also release cytokines, including IFN-γ that activate additional inflammatory cells in the airway. Such nonspecific primary immune responses are essential for early defense against viral infections. At the early stage of the antiviral immune response, dendritic cells (DCs) process viral antigens and then present them to T cells. The representative description of the immune responses against the RSV virus is shown in Fig. 1 as a model for in vitro studies.

### Epithelial cells

The small airways of human lungs consist of mucociliary bronchiolar epithelium and an underlying microvascular endothelium [10]. The small airway epithelium contains ciliated, undifferentiated columnar, club, and basal cells merged to alveolar epithelium with type I (ATI) and type II (ATII) pneumocytes (Fig. 1). ATI cells are the major component of the thin air-blood barrier comprising approximately 95% of the alveolar surface area. The ATII cells cover approximately 4% of the alveolar surface but constitute 15% of all lung cells [11, 12]. Both epithelial cell types are known as important effector cells in inflammatory responses as the location of these epithelial cells increases the chances of ATII cells to encounter a pathogen [13]. Upon exposure to respiratory viruses, ATII cells release several cytokines and chemokines which trigger the migration of monocytes and macrophages to the site of infection. Moreover, human ATII cells also expresses MHC class II molecules on their surface and have been shown to present antigens to CD4^+^ T cells [14, 15]. The resident stem cell population of the lung, known as basal cells, remains near the basement membrane and maintains the ability to replace goblet and club cells [16]. Goblet cells secrete mucus, a key feature of the respiratory immune system as being the first line of defense by trapping bacteria and dust particles before they move further into the alveoli. Club cells express uteroglobin, a vital anti-inflammatory protein, as well as provide physical barrier functions to protect the alveolar airspace [17].

Epithelial cells are widely used in culturing 3D-HTLM. Squamous ATI cells perform gas exchange, and cuboidal ATII cells are critical for immune response by producing surfactants and metabolizing drugs. ATII cells are also able to differentiate into ATI cells when necessary. These airway epithelial cells also provide a barrier function to maintain polarity and alveolar airspace by forming tight junctions [18]. The small airway epithelial cells are critical to understanding immune responses to viral infections in the lower respiratory system. These cells express toll-like receptors (TLRs)-2, 3, 4, and 9, as well as produce cytokines IL-1β, IL-1α, TNF-α, and IL-6 and chemokines GRO (CXCL1), ENA-78, (CXCL5), IL-8 (CXCL8), MIP-2 (CXCL2), NAP-2 (CXCL7), RANTES (CCL5), and MCP-1 (CCL2) [19]. Normal human bronchial epithelial (NHBE) cells from tracheal and carinal biopsies differentiate into ciliated, non-ciliated, and basal cells and have been used to study influenza A and RSV [20].

Small airway epithelial cells express some key markers such as club cell 10 (CC-10), cytokeratin 14 (CK14), prosurfactant protein C (pro-SPC), and multiple aquaporin markers. However, they can be difficult to isolate and culture for long periods and require an air–liquid interface for differentiation. Primary human small airway epithelial cells (HSAECs) from the distal portion of a lung form monolayers and secrete mucus, as well as upregulate cytokine secretion [21]. Upregulation of cytokines IL-1β, IL-6, IL-8, and IL-10 mimics the cytokine storm observed during viral infections in vivo. SP-C, a surfactant released by ATII cells, was downregulated after infection with the Influenza A virus (IAV), confirming the immunogenic effects of the H1N1 virus. Upregulation of surface marker proteins such as aquaporin 5 and cytokeratin 14 was observed during IAV infection, demonstrating the ability of primary epithelial cells to mimic viral infection in vivo [22]. ATI and ATII cells infected with H1N1 and H5N1 strains of IAV secreted cytokines TNF-α, IFN-β, IL-6, RANTES, MCP-1, and IP-10. A diverse representation of small airway epithelial cells is beneficial for developing a complete lung model and expressing cytokines that can be seen in an in vivo immune response to viral infections.

Primary human airway epithelial cells isolated from the bronchioles were infected with RSV-A strain [23]. The value of utilizing primary epithelial cells is noted in the study, which observed the benefit of the inflammatory response of a primary epithelial cell layer and markers that are expressed during a viral infection. RANTES was measured both with and without infection, and upon RSV infection. Through multiple studies, both primary small airway epithelial and primary human bronchiolar epithelial cells have proven the ability to mimic in vivo differentiation. In addition, primary cells used in these models were able to express characteristics as seen in vivo when successfully infected with both HRV-C and HBoV [24].

Since primary epithelial cells are difficult to maintain in culture conditions for extended periods of time, researchers have used different epithelial cell lines. For example, Rajan et al. have developed an in vitro model by infecting the airway epithelial cell line Calu-3 with human rhinovirus (HRV 14 and 16; MOI 0.5) and subsequently exposed to peripheral blood mononuclear cells (PBMC) [25]. The cell line was able to demonstrate an immune response, which peaked in the infected cells on days 3–5. There was an increase in levels of IL-28A, IFN-α, MCP-2, and MIP-1β and a decrease in IP-10, IL-6, and ENA-78 for one or both virus strains. Sundstorm et al. have used a human bronchial epithelial cell line 16HBE14o to culture a 3D air-exposed organotypic human lung tissue model. The 3D model infected with Andes hantavirus was able to demonstrate an immune response, which showed an increase in levels of IL-28A, IFN-α, MCP-2, and MIP-1β and a decrease in IP-10, IL-6, and ENA-78. After infection, cytokines IP-10, IL-6, and IL-8 were upregulated, while RANTES was downregulated, demonstrating a similar immune response found in vivo [26]. Another study by Berg et al. has utilized a human alveolar A549 cell line to obtain 3D models with even distribution of cells across a biomimetic surface. This bio-printed model was successfully infected with influenza A virus, resulting in a clustered infection pattern which can be observed in natural lungs. Further, the bio-ink supported viral replication and the release of pro-inflammatory cytokines, which demonstrated the advantage of a 3D bio-printed model [27]. However, the A549 is a representative cell line of ATII cells and does not contain the ability to differentiate into ATI cells. Additionally, the only cytokine measurement after infection was IL-29, an indicator of the number of virus-infected cells.

Epithelial cell lines have been able to grow as spheroids to study common viral stimulants such as lipopolysaccharide (LPS), an agonist for TLR-4. A549 cells were grown in conjunction with HepG2 cells and activated with LPS [28]. Both cell line cultures were initially grown in 2D, then detached and added to 6-well plates where spheroids were formed using gyratory shaking techniques. While primary cells may be the closest to mimic in vivo conditions, cell lines demonstrate some metabolic activity as primary cells that can be used for models with mechanical stress-loaded environments in which primary cells are not able to survive. For immunological studies, cell lines are not able to demonstrate differentiation or in vivo characteristics that primary cells can have in 3D models [29]. Table 1 summarizes the comparison of different epithelial cells used in 3D tissue models to study various viruses.

**Table 1:** Comparison of epithelial cells used in 3D tissue models to study viruses

Source	Pathogen	Cytokine/protein expression	Infectivity	References
Human primary small airway	Influenza A	Aqp5^−^, CK-14^+^, SP-C^−^^,^ IL-1β^++^, IL-6^++^, IL-8^++^, IL-10^+^, MCP-1^−^	Not measured	[21, 22]
Human primary ATI and ATII	Influenza A	IFN-β^+^, IL-6^+^, RANTES^+^, MCP-1^+^, IP-10^+^	ATI—H5N1 and H1N1: ~ 10^5^ TCID_50_/mL (48 h peak) ATII—H5N1 and H1N1: ~ 10^6^ TCID_50_/mL (72 h peak)	[22]
NHBE	HRV-C HBoV	TNF-α^+^, IP-10^+^, IL-6^+^, IL-8^+^	Not measured	[24]
Calu-3	RSV HRV 14, 16	RANTES^+^ Without PBMC: FGF-Basic^+^, IL-15^+^*, IP-10^+^, IL-6^+^, ENA-78^+^, MIP-1β^−^ With PBMC: FGF-Basic^+^, IL-15^+^*, IP-10^+^, IL-6^+^, MIP-1β^+^*, IFN-α^+^*, MCP-2^+^, IL-28A^+^*, ENA-78^+^	10^6^ RSV-A genome copies/mL (Peak on day 15) HRV 16: 0.04 1/Ct (peak on day 4) HRV 14: 0.07 1/Ct (peak on day 5)	[25]
16HBE14o^−^	ANDV	IP10^+^, IL-6^+^, IL-8^+^, RANTES^−^	50,000 FFU/mL (peak on day 15)	[26]
HCC38	MV	Not measured	100% GFP + epithelial cells four days p.i	[64]
A549	LPS IAV	IL-6^+^, IL-8^+^ IL-29^++^	Not measured ~ 1 × 10^7^pfu/mL IAV titer at 24 h p.i	[28] [27]

AQP5, aquaporin5; CK-14, cytokeratin-14; SP-C, surfactant protein-C; IL, interleukin; MCP, monocyte chemoattractant protein; IFN, interferon; RANTES, regulated on activation, normal T cell expressed and secreted; IP-10, interferon gamma-induced protein-10; HRV-C, human rhinovirus-C; TNF-α, tumor necrosis factor-α; HBoV, human bocavirus; RSV, respiratory syncytial virus; Calu-3, cellosaurus cell line; PBMC, peripheral blood mononuclear cell; FGF, fibroblast growth factor; ENA, epithelial neutrophil-activating protein; MIP, macrophage inflammatory protein; ANDV, andes orthohantavirus; HBE14o, human bronchial epithelial cell line isolated; MV, measles morbillivirus; LPS, lipopolysaccharides; IAV, influenza A virus; PFU, plaque-forming unit

^±^  = significant difference (*P* < 0.05) from control group; ^+±−^ = highly significant difference (*P* < 0.001) from control group, ^+^* = HRV 14 only

### Endothelial cells

The typical mode of infection for respiratory viruses is through the apical surface, thus initially infecting the epithelial layer. However, microvascular endothelial cells play an important role in the productive replication of highly pathogenic IAV. It has also been demonstrated that endothelial cells are the key player in the immune response against IAV [6], being the replication site for influenza viruses [30]. Endothelial cells isolated from small lung vessels infected with IAV demonstrated successful propagation of two different strains of the virus. α-2,3-Sialic acid (SA) and α-2,6-SA receptors were found on the surface of endothelial cells, indicating the susceptibility of influenza virus infections of human pulmonary endothelial cells. Endothelial cells expressed IFN-β, IL-7, TNF, CCL2, and common inflammatory markers such as ICAM1 and VCAM1 after infection with IAV H5N1 as compared to the H1N1 virus. Therefore, influenza viruses can successfully infect and replicate through the endothelial cells, even though they may not be the initial target of the influenza virus [31]. Further, H5N1 has successfully infected and replicated in the endothelial cells co-cultured with ATI epithelial cells from both the apical and basolateral sides [32].

Goodwin et al. have used a rotating wall vessel (RWV) to develop lung tissue-like assemblies that contain primary human bronchio-tracheal cells (HBTC) and a transformed human bronchial epithelial cell line (BEAS-2B) [33]. These cultures were then infected with various viruses like SARS-CoV, RSV, and HPIV3. Although the immune response to the added viruses was not recorded, future studies can focus on the functionality of cells developed in the 3D tissue-like assemblies. Unfortunately, very few studies have reported the use of 3D models that include pulmonary microvascular endothelial cells. Seeing their importance in the immune response of the lower respiratory system, it would be crucial in future studies to incorporate endothelial cells to mimic in vivo-like conditions.

### Myeloid cells

An important component of the immune response in the lungs comes from hematopoietic origin. These cells consist of neutrophils, monocytes, macrophages, mast cells, dendritic cells (DC), and lymphocytes [34]. Mast cells, dendritic cells, and macrophages are lung resident cells, while neutrophils, monocytes, and lymphocytes are recruited through cellular signaling during lung infection. Once recruited, monocytes can differentiate into resident macrophages derived by the signaling of lung parenchymal cells (epithelial cells, endothelial cells, and fibroblasts). There are two types of pulmonary macrophages: alveolar macrophages, and interstitial macrophages. Both types of macrophages are recruited into the lung parenchyma and release anti-inflammatory cytokines, which restrict inflammation and promote tissue repair [35]. Macrophages exposed to inflammatory stimuli begin secreting cytokines such as tumor necrosis factor (TNF), IL-1, IL-6, IL-8, and IL-12 [36]. Dendritic cells aid in the recognition and processing of antigens. Therefore, DC act as a part of the adaptive immune system. Since monocytes have the capability of migrating and differentiating into macrophage and dendritic cells, they play an important part in evaluating the immune response to viral aggregates in human lung models.

DC are antigen-presenting cells crucial in the initiation of immune responses to viral infections. In humans, the DC circulating in blood have been classified as myeloid DC (MDC) and plasmacytoid DC (PDC) [37]. MDC are of myeloid origin expressing CD13, whereas PDC are derived from lymphoid progenitors and express T- and B-cell molecules [38]. Dendritic cells and cells derived from peripheral blood mononuclear cells (PBMC) are included in co-cultures with epithelial cells to study immune responses to pathogens [21, 39]. One of the main focuses of adding PBMC to a co-culture in 3D tissue models is to examine the differentiation of mononuclear cells in response to specific viruses. PBMC grown in co-culture with airway epithelial cells and cytokines, such as GM-CSF and IL-4, demonstrate the differentiation and maturation of monocytes into mature DC. Migration of the DC to the epithelial layer and DC derived from monocytes demonstrate many in vivo-like qualities that can be used to study additional viral infections. In another study, PBMC were grown in co-culture with epithelial cells to examine if IP-10 and IFN-α were upregulated after infecting the apical side of the epithelial cells with human rhinovirus [25]. The effect of PBMC was quantified by the downregulation of IL-6, IP-10, and ENA-78 and upregulation of IL-28A, IFN-α, MCP-2, and MIP-1β. PBMC are vital in modeling viral pathogenesis in lung models due to their ability to differentiate into resident macrophages as well as DC. DC have demonstrated viral replication and antigen recognition as a part of the immune response within the lung.

## Scaffolds used in 3D tissue-engineered lung models

The scaffolds used in a 3D model must have the appropriate chemical and physical properties to effectively simulate in vivo conditions. A variety of materials are used to construct 3D scaffolds. These materials encompass the extracellular matrix or the space that the cells move through. They must be biocompatible so that the cells can grow and differentiate naturally in these scaffolds. Given the correct conditions and signals, resident cells added to an extracellular matrix will form multilayered tissue structures. The most important characteristics of a scaffold for a tissue model is its ability to maintain cell-recognizable surface chemistries, mechanical integrity and the ability to induce signal transduction [40]. To create a biomimetic environment for a lung model, the supporting scaffold must be conducive for gas exchange as well as maintain a strong barrier function to preserve the alveolar airspace. Porosity is another integral component necessary for mimicking the recruitment and migration of resident alveolar macrophages from PBMC in the lower respiratory system. The scaffold should support high seeding efficiency and proliferation, as well as support the viability of cells. For the 3D-HTLM, the ideal thickness of the scaffold should be around 2 μm [41]. Further, the presence of collagen fibrils is necessary for the stress/strain cycles of alveolar tissue faced during respiration [42]. The scaffold types that have been utilized for lower respiratory lung models include natural polymers, such as collagen, alginate, nutrient rich Matrigel, as well as decellularized lung tissue.

### Collagen matrix

Collagen is the most used scaffold to make a soft, porous hydrogel. Collagen accounts for approximately 25% of dry mass in mammals, making it one of the most abundant proteins found in the body [43]. Therefore, it is an easily acquired and well-defined matrix. In addition, collagen has weak antigenicity and high biocompatibility, making it an attractive option for the development of tissue models. Extracellular matrix (ECM) collagen is produced when fibroblasts are added to the cultures, which aid in organizing collagen into fibrils, lending to additional structural integrity. Additional proteins can be mixed with collagen to strengthen the gel, making it capable of supporting a larger system of cells. For example, Bhowmick et al. mixed chitosan, a naturally occurring protein, with collagen to study IAV in a human small airway epithelial model [21]. Collagen was cross-linked with chitosan, which gave the scaffolds suitable mechanical properties for housing and maintaining lower respiratory tract cells. This produced a porosity of 70.3 ± 4.3% and an average pore area of 1000 μm^2^, which allowed cellular organization similar to in vivo [44]. However, collagen alone has been found to provide enough structural integrity to support 3D growth culture. Collagen combined with DMEM seeded with MRC-5 cell line supported epithelial cell line 16HBE14o- infected with ADV for at least 39 days, demonstrating the long-term structural integrity of pure collagen by maintaining the viability of cells for extended periods [26]. It can also be used as a bio-ink with the use of 3D microextrusion printing technology to engineer the collagen fiber alignment, as it has been demonstrated to successfully grow epithelial cell clusters [45].

### Alginate

Alginate is a naturally available polysaccharide derived from the cell walls of brown seaweed. It is known for its durable mechanical properties in hydrogels and its biocompatibility with mammalian cells. However, alginate can block cell adhesion due to its carboxyl groups which give it an overall negative charge [46]. Soaking alginate in collagen makes it more biocompatible for cellular attachment and proliferation. For instance, iPSC-derived mesenchymal stem cells grown on 3D alginate beads with fetal lung fibroblasts successfully grew as organoids [47]. The alginate-based organoids were also formed in a rotating wall vessel bioreactor, which arranged the molecules as alveolar cells just like in vivo. Stem cells formed tissue-like assemblies which closely resembled in vivo conditions, indicating the potential for alginate bead-grown cell systems with co-cultured cells. Polymerizing alginate beads with a high concentration of rat collagen solution aided the cellular attachment and growth within the 3D tissue models. These 3D tissue models could have potential future applications in pathogenic studies; however, it faces complications of the long-term viability of the system due to the intrinsic cells in the center of the beads [48]. Alginate beads are also not able to mimic the recruitment and differentiation of immune cells because of the lack of a flow through multilayers*.*

Alginate, Matrigel, and gelatin mixture were used to make a 3D tissue model by using a 3D printed bio-ink. Every aspect of the scaffold is controlled down to the pore size when utilizing high-quality 3D printing, which can be optimized for nutrient delivery. A549 cells were seeded in the platform, then a seasonal strain of Influenza A was added to the scaffold to observe the behavior of the biomimetic model [27]. The high precision of the 3D printing technique resulted in a distribution of the virus and cells similar to in vivo. With 50% Matrigel concentration in the alginate bio-ink, the cells remained evenly distributed throughout the models. However, the model remained viable for only seven days [49]. Both alginate beads and alginate-based bio-ink display an even cellular distribution, a key feature in modeling alveoli, as well as successfully supporting co-cultures of cells. Alginate, along with other natural polymers, is not immunogenic, which makes them ideal for pathogenic research. Wilkinson et al. also demonstrated that alginate-based scaffolds allow cells to maintain their phenotypic characteristics as observed in vivo [47]. However, specialized equipment was required in using alginate for pathogenic research, which is not inherent to this scaffold type. But a more simplistic approach in the future could demonstrate an accessible way to utilize the material.

### Matrigel

Matrigel is a solubilized basement membrane matrix secreted by murine sarcoma cells and resembles the laminin and collagen IV-rich extracellular environment found in many tissues. Like other natural materials, such as alginate, collagen, and gelatin, it forms a gel-like structure that supports the cells to make a 3D tissue platform. Matrigel contains collagen, laminin, and other important extracellular proteins and growth factors. This makes it a desirable material for constructing a scaffold to mimic the extracellular matrix. Matrigel is also biocompatible and supports the attachment and proliferation of cells. It is mainly utilized in studies containing stem cells since it behaves as a basement membrane, where stem cells typically attach [50]. However, a disadvantage is that the contents are not fully characterized or uniform from batch to batch, introducing additional variability into each system.

Lung organoids are naturally developed three-dimensional structures showing physiological features of the lung as well as cell–cell interactions. Stem cells mixed with different lung cells from an explant, when cultured in Matrigel scaffold, grow into lung bud organoids. Chen et al. have shown that embryonic stem cells successfully differentiated into ciliated epithelial cells when Matrigel was enriched with branching medium [51]. The organoids maintained morphological and phenotypical features of the epithelial cells similar to in vivo*,* showing the biocompatibility and biomimicry of Matrigel [52]. To further demonstrate its ability to grow and support human airway epithelial cells, Matrigel was used as a nutrient source to develop “alveolospheres” consisting of ATII cells to propagate SARS-CoV-2 infection. The alveolospheres successfully mirrored immune response features observed in COVID-19 lungs [53]. These featured studies have shown that Matrigel successfully supported the development of stem cells into differentiated airway epithelial cells, despite its intrinsic variability among models.

Matrigel grown organoids infected with IAV have shown that some strains of avian influenza replicated better than others. The kinetic rate of infection varied among epithelial cell types, indicating replication kinetic dependency on cell type rather than scaffold material [54]. More kinetic modeling of this disease would be beneficial for measuring the differences using stem cells versus tissue explant in the future. In another study, a 3D Matrigel model was used to mimic the distal lung in vivo. Murine-adapted Influenza A injected into the scaffold to observe the differentiation and dispersion of p-63 stem cells. The stem cells developed into alveolar-like cells, and they agglomerated around damaged regions of parenchyma post-infection. Cytokines associated with alveolar cells were also detected in the scaffold, supporting that the desired differentiation was achieved from this model [55].

Matrigel-based 3D tissue models have been successfully developed to study the viral pathogenesis of parainfluenza type 3 (PIV3) and recombinant measles virus (MeV). Human airway epithelial cells and pluripotent stem cells were grown in the Matrigel before inoculation to mimic the distal lung response in infants. Over time, the structure resembled that of the distal lung in vivo, including stem cells that behaved like alveolar cells. In the PIV3 model, no damage occurred to the tissue post-infection, but the virus proliferated throughout the structure similar to in vivo. Furthermore, the MeV model underwent syncytia and sloughing of epithelial cells, which also occurs in vivo [56]. Similarly, Matrigel-based organoids from donor-derived airway epithelial cells can be successfully infected with H7N9 and H1N1 influenza virus strains [57]. After growth and differentiation within the Matrigel, ciliated epithelial cells demonstrated ciliary beats, which aided in the propagation of the influenza virus strains. In addition, a 3D culture of human airway epithelium in Matrigel demonstrated Rhinovirus C and Bocavirus infections that were not seen in monolayer cell culture or with certain cell lines, possibly due to the lack of ciliated cells [24]. Organoids have also demonstrated similar replication kinetics and cellular diversity as ex vivo lung tissue explants when infected with IAV and H1N1 [52]. Results from these multiple organoid studies display the biocompatibility of Matrigel to develop lung organoids for studying viral pathogenesis and immune responses in the respiratory system.

### Decellularized tissue

The benefit of using a decellularized tissue is that it has the natural structure found in vivo. This natural structure is more suitable for the habitation of cells and retains the vascular network. These models are the most mimetic of in vivo conditions. However, decellularized tissue can cause an immune response once cells are loaded onto the tissue, making it difficult to characterize immune responses to specific viral pathogens. At first, the process of removing cells and other non-structural biological components from tissue can be lengthy and damaging. However, with recent advances, tissue can be decellularized within 24 h and still maintain original structural integrity to effectively house the cells [58]. While acellular human lung tissue is ideal for repopulating with human cells, large quantities of donor tissue can be difficult to obtain. It has been shown both murine and porcine tissue is a viable alternative for decellularized tissue to re-populate with human cells for studying lower respiratory pathogens [59, 60]. While other animal tissues may be a viable substitute, human tissue remains the ideal candidate for decellularized tissue.

Ghaedi et al. have implanted induced pluripotent stem cells (iPSCs)-derived AT1 and AT2 cells into acellular lung and have shown successful differentiation of pulmonary epithelium [61]. Other studies use acellular tissue as a scaffold to study immune responses to various pathogens. Gilpin et al. found that neonatal lung ECM contains FBN-2 and TN-C, proteins that participate in lung development and repair [62]. Dorrello et al. have developed an airway-specific approach to remove the pulmonary epithelium, while maintaining function of the vascular endothelium in a rat model. The resulting vascularized lung grafts supported the attachment and growth of human adult pulmonary cells and stem cell-derived lung-specified epithelial cells [63]. In another study, decellularized porcine small intestinal submucosa was seeded with human fibroblasts and epithelial cells before infection with measles. The migration speed of dendritic cells was measured as a function of several variables, and cytokines associated with epithelial immune response were detected, indicating measles is replicable in the decellularized tissue for disease modeling. [64]. Future studies are likely to focus on bioengineering of functional lungs by utilizing decellularized lung tissue, whether from human, murine, or porcine origins, as it is promising to not only the tissue replacement in vivo but also study viral pathogenesis of lung.

## Culture conditions and biophysical features for 3D tissue-engineered lung models

One of the biggest challenges of creating a fully biomimetic tissue model is developing ideal cell culture conditions. In the human body during a pathogenic invasion, cellular responses can signal for immune responses through the upregulation and downregulation of certain cytokines and chemokines. Studies have shown differing behavior in cells grown in varying conditions, such as co-culture, 3D culture, air–liquid interface (ALI), and flow conditions. It is important to establish the significance of which culture conditions are necessary to include in a tissue model to fully understand the pathogenesis and immune response. For instance, alveolar macrophages signal anti-inflammatory proteins such as SOCS1 and SOCS3 to the epithelial layer during infection, possibly inhibiting damage to the epithelial tissue, so a co-culture is needed to mimic the in vivo immune response [65]. In the human lung, the epithelial layer is exposed to the air for oxygen uptake and delivery, so maintaining ALI can help mimic alveolar physiology. Bioreactors have recently been used to introduce flow conditions, but due to the high sensitivity of lung models, few studies have been able to create and maintain these models. Considering these culture conditions in developing a lung model can help build an ideal model for a better understanding of viral pathogenesis, and ultimately how to treat or prevent these infections.

### Co-culture conditions

A key feature of studying the immunological response to pathogens is the use of co-culture systems. Parenchymal lung cells such as epithelial cells, endothelial cells, or fibroblasts can be co-cultured with immune cells such as macrophages or DC. Co-culture systems not only allow the study of immune responses, but also help to mimic in vivo physiological conditions [64]. A triple co-culture of fibroblasts, epithelial cell line H358, and monocytes were grown in decellularized porcine tissue. This system showed successful differentiation of monocytes into DC, and epithelial cells developed into a dense cell multilayer on the apical surface, while fibroblasts migrated to the connective tissue [64]. Sundstorm et al. have utilized a co-culture system of fibroblasts and epithelial cells infected with Andes Hantavirus resulting in successful infection and propagation of the virus throughout the model. Epithelial cells demonstrated a typical in vivo-like immune response*,* which included upregulation of pro-inflammatory cytokines such as IP-10, IL-6, and IL-8 [26]. Another study has reported the co-culture of lung fibroblasts and epithelial cells to model idiopathic pulmonary fibrosis by stimulating the organoids with TGF-β1 [47]. Both studies have shown the interaction of cells with one another to propagate and model diseased conditions of the lungs. Therefore, co-culture models are useful to understand the pathogenesis of the introduced viruses.

### Three-dimensional cell culture

Three-dimensional cell culture is defined as cells grown on or within a 3D matrix, where cells and solutes are able to interact each other within three dimensions. Characteristics of human primary cells such as cell viability and cell protein expression were altered when cells were grown on a 3D scaffold [66]. Epithelial cells grown on a 3D matrix have shown viral replication similar to in vivo. This could be due to the microspheres and cilia developed in the 3D culture that mimic alveolar epithelial layer physiology. Common epithelial markers pan-cytokeratin (PCK), CK5, and ZO-1 were expressed by the 3D model of the alveolar epithelium. Further, the 3D epithelium showed a marked upregulation of TNF-α, IP-10, IL-6, and IL-10, after HRV-C and HBoV infection [24]. Similar findings were observed when 3D spheroids of epithelial cell line A549 were stimulated with LPS. The stimulated spheroids have shown significant upregulation in cytokine expression for IL-6 and IL-8 [28]. Furthermore, Berg et al. printed and infected 3D lung models of A549 cell line with IAV. IAV was found to be clustered in 3D conditions, which closely mimics the biological condition of infection [27]. These findings agree that 3D models of primary epithelial cell culture are crucial in developing models for in vivo-like immune responses against viruses.

### Air–liquid interface

Epithelial cells are accustomed to being exposed to air in vivo, thus simulating this effect in culture is critical. Air–liquid interface (ALI) is a term used to describe when cells are exposed to air on the apical surface, while the bottom layer (basolateral surface) remains submerged in a liquid medium. Due to this key physiological feature, ALI exposure is necessary for epithelial cells to respond in a way that mimics their actions in vivo. ALI aids in the differentiation of epithelial cells into the resident cells typically found in the human lung, such as ATI, ATII, goblet, club, and ciliated cells, each of which performs specific duties of interest necessary for their functionality [67]. Epithelial cells grown at ALI have been shown to fully differentiate into pseudostratified epithelium, modeling a confluent layer of the small airway apical surface [24]. Further, ALI exposed epithelial cells showed the upregulation of IL-6, IL-8, and IP-10, along will eotaxin-1 and VEGF-A after infection with HNDV [26]. Epithelial cells grown within a 3D model exposed to ALI have demonstrated 25.6% higher viability than those grown in non-ALI culture [21]. Morphologically, the epithelial cells were highly comparable to in vivo cells both before and after infection with IAV.

**Fig. 2:**
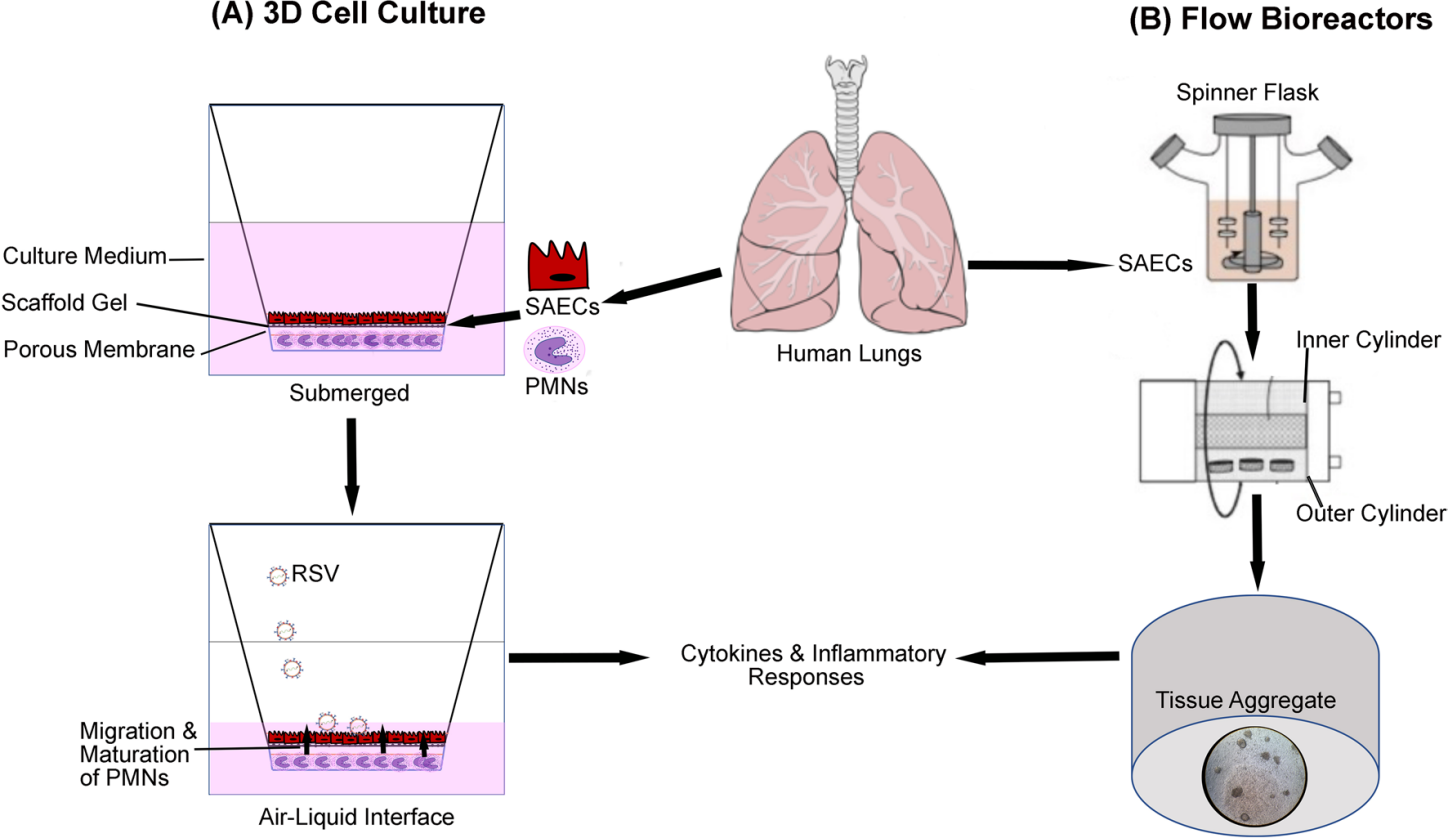
Schematic presentation of three-dimensional in vitro lung tissue models to mimic immune responses following RSV infection. **A** Developing a 3D model within a hanging cell culture inserts with a porous membrane allows for nutrient exchange when exposed to air–liquid interface. Epithelial cells grow on the surface of the membrane and form tight junctions, while immune cells such as polymorphonuclear (PMN) leukocytes remain submerged. **B** Flow bioreactors using small airway epithelial cells (SAEC) to grow tissue aggregates infected with viruses. The release of cytokines and chemokines can be measured from the collected media

To standardize the development of the epithelial layer at ALI, certain products have been developed to grow the epithelium for viral research. For instance, MucilAir™ and SmallAir™ are used to develop airway epithelium containing ciliated, goblet, and basal cells. MucilAir™ is used to model the upper airway epithelium, and SmallAir™ mimics the small airway epithelium. These platforms utilize differentiated nasal epithelial cells that secrete cytokines and chemokines, produce mucus, and have mucociliary clearance. MucilAir™ validated the immune response of the tissue-engineered scaffold after inoculation of picornaviruses, IAV, and a type of coronavirus [68]. Further, it has shown that antiviral injections inhibit the behavior of the viruses introduced to the MucilAir platform. In a subsequent study, MucilAir™ medium was used with a porous culture insert containing airway epithelial cells to model RSV-A infection [23]. ALI was maintained during RSV infection and inhibitor testing. Epithelial cells produced mucus and displayed ciliated beating. The antiviral infusion inhibitors provided only initial relief for the system; however, nucleoside and non-nucleoside replication inhibitors proved to robustly reduce viral load at all time points throughout the study. Both studies that utilized MucilAir™ demonstrate similar responses and characteristics found in epithelium developed at ALI. It is evident that epithelium benefits from growing at ALI, but it may be hard to streamline production due to the careful attention needed to maintain ALI. Cohesively, these studies demonstrate the necessity of maintaining lung epithelial cells at ALI to mimic in vivo conditions for differentiation and successful viral infections. A schematic representation of ALI cultures condition to study immune responses is shown in Fig. 2A.

### Flow/bioreactors (liquid and gas)

Lung tissue engineering has seen a rapid growth of bioreactor development at both microfluidic scale to the human-sized lung [69]. Bioreactors are used to simulate in vivo conditions, producing a physical stimulus for cells to respond, as well as monitoring and reacting to cell growth conditions. Both gas and liquid bioreactors are used for this application. Tuning the flow rate of a bioreactor is key to mimicking physiological conditions. If the flow rate is too high, the scaffold might get damaged by sheer force or cells could be pulled from the scaffold. If it is too low, it will not be effective at producing the stimulation necessary to elucidate the desirable response from the cells [70].

For lung models, rotating wall bioreactors are mainly used for organoid or microsphere development and maintenance (Fig. 2B). A 3D alveolar model utilized a rotating wall vessel bioreactor in the development of lung and neuronal tissue scaffolds [33]. In this bioreactor, a cylinder was filled with medium, and primary human bronchio-tracheal cells (HBTC) were suspended in a solution with Cultispher G microcarriers. When the bioreactor wall rotates, the liquid circulates at the same speed, and the cells seeded in the microcarriers proliferate under these conditions. Oxygen can diffuse through the medium to provide air for the cells with negligible turbulence and shear force. A normal human bronchio-tracheal cell line was then added to the bioreactor, followed by normal human neuronal progenitor cells. Agglomerates of tissue-like assemblies (TLA) form about the microcarriers, creating a 3D structure. Different viral loads of PIV3, RSV, SARS, and VZV were injected into the platform to observe immunogenicity. The results have shown a successful inoculation and viral budding of both RNA and DNA viruses. The tissue model has sustained various viruses for several weeks in the bioreactor by simply replenishing media.

Another rotating wall bioreactor was used for drug screening of lung diseases. In this bioreactor, mesenchymal stem cells were seeded in interstitial spaces between alginate beads from an electrostatic droplet generator. Later, fetal lung fibroblasts were added with medium and alginate beads to a rotating wall bioreactor. Stiff organoid structures were obtained and moved to trans-wells, after which they were placed on a laboratory rotisserie to finalize structural growth. The organoids were modeled after distal lung alveolar sacs, and the process can be easily upscaled for high throughput disease modeling. The synthesis of organoids is highly optimized, allowing for manipulation of bead stiffness, composition, and size as well as cell type and number [47]. Although both studies have used rotating wall bioreactors for 3D model development, Wilkinson et al. were able to develop organoids while Goodwin et al. created microspheres. Due to the spherical nature of both types of models, the utilization of bioreactors can be studied in the future for cylindrical, plated 3D models.

Few studies have utilized bioreactors for studying viral infection within 3D lung models due to the high sensitivity of the models, such as maintaining ALI and the delicate nature of the epithelial layer. However, for efficient production purposes of lung models for disease research, it is a potential area for improvement. Therefore, it would be beneficial for future research to incorporate this aspect for related experiments. This would add another dimension of complexity to the 3D-HTLM that would promote a physiological response from cells. It would also promote the possibility of screening diseases on a larger scale for precision medicine or patient-specific models.

## Conclusions

The area of 3D in vitro models is a promising field of research for studying lower respiratory viruses with some drawbacks to overcome. The literature has shown that 3D in vitro HTLM is promising for the study of viral pathogenesis. These models can be more predictive of treatment strategies when transitioning from experimental to clinical trials. Lung organoid cultures have been utilized in many studies to investigate the tropism of viruses; however, they cannot apply viruses to the apical surface due to the intrinsic epithelial layer formed. Further, organoid cultures require feeder cells or Matrigel for long-term maintenance in vitro. Researchers are using single-cell transcriptomic analysis to prepare cell culture media having relevant growth factors. Another promising area that has not been as widely explored is decellularized lung tissues repopulated with primary human cells. Decellularized human lung tissue could be the most mimetic model since it provides the same scaffold composition as in vivo conditions and thus allows for accurate studies of migration and pathogenesis of infectious agents.

Utilizing co-culture conditions in lung models provide a more in vivo-like immune response when introduced to viruses through the emission of unique cellular signaling proteins and molecules. One difficulty to overcome with co-culture systems is developing a singular growth media to support the growth of all cell types and maintaining ALI for the development and differentiation of epithelial cells into a confluent epithelium layer. One possible solution is to use a bioreactor that regulates the medium level in a hanging cell culture well insert plate. Recently, Ingber and his team at the Wyss Institute for Biologically Inspired Engineering at Harvard University has created lung-on-a-chip. The airway chips are made of a clear, flexible polymer and contain two parallel microchannels separated by porous membrane. One of the channels is for fluid, and the other one is for air. The primary human airway tissue cells are cultured on the porous membrane. These cells multiplied and matured into specialized cell types after 2–3 weeks. Lung cells grow on the air side of the membrane, and cells that line blood vessels grow on the other with a pump moving culture medium through the fluid channel. Some of the lung cells in the airway chip create mucus and even grow cilia, tiny hairlike structures that move mucus and help protect the body from infection. A pseudo-virus was used to study the initial stages of infection and viral entry into lung cells. Further, the airway chips have been used to screen various drugs for use against influenza and SARS-CoV-2 [71].

A reliable mechanistic approach to maintain an air–liquid interface is crucial aspect of studying immune responses against viral infections. However, culturing and developing 3D in vitro models require extensive training and can be tedious to obtain few results, which limits the throughput of the systems. Therefore, feeder-free culture, bioreactors such as rotating wall vessels and lung-on-a-chip to develop tissue-like assemblies may be necessary for scale-up production of HTLM. Overall, 3D in vitro HTLM shows promise to be the next frontier of disease research, especially immune responses due to lesser variability, ability to translate to clinical trials, and promote cell–cell interactions as seen in vivo.

## Data Availability

Not applicable.
